# Maslinic acid derived from olive fruit in combination with resistance training improves muscle mass and mobility functions in the elderly

**DOI:** 10.3164/jcbn.18-104

**Published:** 2019-03-07

**Authors:** Narumi Nagai, Satomi Yagyu, Anna Hata, Shinsuke Nirengi, Kazuhiko Kotani, Toshio Moritani, Naoki Sakane

**Affiliations:** 1 School of Human Science and Environment, University of Hyogo,1-1-12 Shinzaike-honcho, Himeji, Hyogo 670-0092, Japan; 2 Division of Preventive Medicine, Clinical Research Institute, National Hospital Organization, Kyoto Medical Center, 1-1 Mukaihata-cho, Fukakusa, Fushimi-ku, Kyoto 612-8555, Japan; 3 Division of Community and Family Medicine, Jichi Medical University, 3311-1 Yakushiji, Shimotsuke-shi, Tochigi 329-0498, Japan; 4 Department of Sports Sociology and Health Sciences, Kyoto Sangyo University, Kamo-honmachi, Kita-ku, Kyoto 603-8555, Japan

**Keywords:** maslinic acid, resistance training, muscle mass, knee pain, geriatrics

## Abstract

Maslinic acid, derived from olive fruit, reduces pro-inflammation cytokines, which are involved in muscle fiber atrophy. Therefore, the maslinic acid ingestion may enhance the muscular response to resistance training through anti-inflammatory action. We therefore conducted a parallel, double-blind, randomized, placebo-controlled trial that examined whether a combination of maslinic acid supplementation and resistance training improve mobility functions in community-dwelling elderly persons. Over a 12-week period, 36 participants underwent moderate resistance training and are assigned to the maslinic acid supplementation (*n* = 17, 60 mg/day) or the placebo (*n* = 19) group. At baseline and at 12-weeks, we assessed body composition, grip strength, walking speed, leg strength, mobility functions, and knee pain scores. Following the 12-weeks, skeletal muscle mass, segmental muscle mass (right arm, left arm, and trunk) and knee pain score of the right leg were significantly improved in the maslinic acid group, while there was no change or parameters had worsened in the placebo group. Grip strength of the better side significantly increased only in the maslinic acid group. These results suggest that maslinic acid supplementation combined with moderate resistance training may increase upper muscle mass and grip strength, and reduce knee pain, could be effective for preventing mobility-related disability in elderly persons. Clinical trial registration number: UMIN000017207.

## Introduction

Japan has the oldest population in the world, and the aging rate is predicted to reach over 40% in 2055,^(1)^ which might be associated with more disability and more dependency. In such aging society, sarcopenia have gained special attention.^(2)^ Generally, skeletal muscle atrophy with aging is inevitable; however, reduction of muscle or muscle strength more than the natural decrease due to aging is defined as sarcopenia, and it is likely to lead to physical frailty and to progress to a need for long-term care.^(3)^ To maintain the daily activity level and health-related quality of life (QOL) of the elderly, prevention of reduction in skeletal muscle and muscle strength is important.^(4,5)^

Both the pathologies of sarcopenia and physical frailty are based on atrophy of muscle fiber, and exercise or nutrition intervention or both can be performed to prevent it.^(3)^ In a systematic review^(6)^ of intervention studies on prevention of disability involving physically frail elderly persons living in the community, only 3 of 10 studies included in the final analysis achieved favorable (positive) results by exercise intervention alone. For the muscle atrophy described above, nutrition intervention by ingestion of amino acids, such as leucine, and protein has been conducted because the atrophy depends on the balance of the protein mass in muscle fiber, that is, between muscle protein synthesis and proteolysis.^(3)^ However, improvement of body composition by nutrition intervention alone in elderly persons was noted only in a very few studies.^(7)^ Intervention combining exercise and nutrition has been reported to be effective to improve the physical function and muscle mass in elderly persons with sarcopenia,^(8)^ however, the efficacy for healthy elderly persons is unclear.

Since inflammatory cytokines are involved in muscle fiber atrophy, ingestion of food components with anti-inflammatory action may inhibit loss of muscle mass. For example, maslinic acid (MA, 2-α, 3-β-dihydroxyolean-12-en-28-oic acid), a natural plant-derived triterpenoid compound, is known to have preventive effects against oxidative stress and pro-inflammatory cytokine generation.^(9,10)^ In an animal study, oral administration of the MA derived from olive fruit suppressed expression of inflammatory-related genes in knee joint tissues.^(11)^ As another non-pharmacological approach, resistance training (RT)^(12)^ and aerobic exercise^(13)^ improved physical performance and the level of inflammatory biomarkers, such as hs-CRP, in patients with type 2 diabetes mellitus. Reducing systemic and muscle-based inflammation may promote muscle hypertrophy and by attenuating muscle atrophy, contribute muscle strength and physical function.^(9)^

Based on the aforementioned findings, we hypothesized that the MA ingestion may enhance the muscular response, such as muscle hypertrophy, to RT through an anti-inflammatory action in elderly individuals. Accordingly, the purpose of this study was to examine the effects of the MA supplementation in combination with moderate RT on muscle mass and the parameters related mobility functions in a parallel, double-blind, randomized, placebo-controlled trial, in community-dwelling elderly persons.

## Materials and Methods

### Study design

This study was carried out from April 21 to August 11, 2015 in a randomized, double-blind, and placebo-controlled design, performed mainly at the Department of Food Science and Nutrition, University of Hyogo in Himeji City, Japan. To determine the effects of 12-week, moderate RT with or without the MA supplementation, participants were randomly assigned to the MA supplementation with moderate RT group (MA group, *n* = 19) or the placebo supplementation with moderate RT group (placebo group, *n* = 20). Participants were allocated to the MA or the placebo group with randomization stratified by age and gender by an independent statistician,^(14,15)^ using permuted-block randomization (block-size: 2) with an allocation ratio of 1:1. This statistician was not involved in participants’ recruitment and any measurements. Both participants and investigators responsible for assessing the outcomes were blinded to the randomization status. On completion of data collection, all the data were fixed. Then, statistical analyses were conducted. During the 12-week intervention period, compliance with the study protocol was assessed at 2-week intervals through the collection of unused supplements and the inspection of daily records of supplement consumption and home exercise (type and duration). All study measurements were performed at the beginning and at the end of the intervention period.

### Participants

Participants were recruited through announcements to second-year attendees of a weekly stretch training program for the elderly at a public liberal arts school in Hyogo prefecture, Japan. Of the 42 interested individuals, 12 males and 27 female subjects (71–76 years) met the study inclusion criteria and volunteered for study participation. According to the inclusion criteria, those aged >65 years who had undergone stretch training for the previous 12 months were included. According to the exclusion criteria, those who received public health nursing care, had any contraindications to RT, or had been diagnosed with dementia by a physician or were undergoing dementia treatment were excluded. Prior to inclusion, all the participants received a detailed explanation of the study and they provided written informed consent. All study procedures were conducted as per the Declaration of Helsinki. The study was approved by the Research Ethics Committee of the School of Human Science and Environment, University of Hyogo (No. 117, April 14, 2015), and was registered at the University hospital Medical Information Network Center in Japan (UMIN000017207)

### Nutritional assessment

Nutritional assessment before intervention was conducted by national registered dietitians using (1) the short version of the Mini Nutritional Assessment (MNA)^(16)^; and (2) a self-report record of all beverages and weighed food consumed on a typical weekday. During the intervention period, the dietitians met almost all participants every week to assess whether or not participants change their dietary patterns or meal frequency, using weekly monitoring seat.

### Products administered

We prepared the jelly containing the MA derived from olive fruit extract (Nippon Flour Mills CO., Ltd., Tokyo, Japan).^(17)^ Participants were instructed to consume daily either 20 g of the MA jelly (60 mg of MA) or the placebo jelly (without MA) preferably after breakfast for 12 weeks. Both experimental supplements included an equivalent amount of water, gelling agent, emulsifying agents, pH adjusters, grapefruit seed extract, sweetener, and aroma chemicals.^(17)^

### Training program

All the participants participated in a RT program at the public liberal arts school every week. This series of training class was supervised by an experienced sports scientist and a Tai Chi instructor. Each training class comprised the following: a warm-up session (30 min); RT [40 min; combination of latex band training (Thera-band^®^, Performance Health, Akron, OH), squat, and Tai Chi]; and a cool-down session (20 min). In addition, participants were encouraged to perform daily exercises at home, such as stretching, squatting, or walking and to record the types and duration of each exercise session.

### Outcome measurements

Anthropometric and blood pressure measurements were conducted at the beginning and at the end of the intervention period, as described previously.^(18)^ To evaluate the body composition, a bioelectrical impedance analyzer (InBody S10, BioSpace, Seoul, Korea) was used.

All physical measurements were examined by health fitness programmers at the beginning and at the end of the intervention period. Grip strength was measured using a digital hand-grip dynamometer (TKK5401; Takei Kiki Kogyo, Niigata, Japan). Low-grip strength is predictive of limited physical function, particularly in older adults; therefore the use of hand-grip dynamometry is a fundamental element of the physical examination.^(19)^ These tests were repeated twice with the dominant hand (better side), and the average value was determined.

A chair-standing test 30 was performed as described by Jones *et al.*^(20)^ The participant was encouraged to complete as many full stands from a chair as possible within 30 s. The score is the total number of stands.

Usual gait speed assessment was performed by a 5-m gait speed test^(21)^ in a hall which was well-lit, unobstructed, and had sufficient space. Gait speed was measured between the central 5 m (3–8 m) within the 11-m line, recorded by a phototube-based speed measurement system (Time recorder from Assist, Co. Ltd., Tokyo, Japan; Reflective photo sensor WT24-2B410, SICK, Co. Ltd., Düsseldorf, Germany). Participants were instructed to stand behind a starting line, and then to walk at their usual pace along an 11-meter line. The timed 5-m walk (in seconds) was calculated from the mean of two trials.

Knee functions and the extent of knee pain were evaluated by using a Japanese self-administered questionnaire based on English version of the Western Ontario and McMaster Universities osteoarthritis index (WOMAC).^(22)^ This questionnaire is consisted of 27 items including: activity of daily living (knee function, 17 questions) and degree of right/left knee pain (5 questions, each). Each question has five subscales from the best to the worst situations. Higher score represents better situations or less pain.

### Sample size

Sample size was calculated using the knee pain score as the outcome. This calculation showed that a sample size of 21 participants per group was required to detect an inter-group difference of 2 points on the knee pain score (power = 0.8, alpha = 0.05) between baseline and 12 weeks (G*Power, ver. 3.1.9^(23)^). This predicted difference equated to a large effect size of 0.8 or greater.

### Statistical analyses

All measurements were expressed as means and SD values. All statistical analyses were conducted using the Statistical Package for the Social Sciences (SPSS for Windows^TM^ ver. 22, IBM Inc., Tokyo, Japan). Prior to statistical evaluation, normality testing was conducted using the Kolmogorov-Smirnov test. Baseline differences between the MA and placebo groups were tested using unpaired *t* tests. Intra-group changes in the values between baseline and 12 weeks were tested using paired *t* tests. To determine whether measurements were influenced by MA supplementation, two-way analysis of variance with repeated measurement was conducted. Here, the factors were group (MA or placebo) and time (baseline and 12 weeks). Effect size of the mean differences was determined using Cohen’s *d*. The magnitude of the effect size was determined by Hopkin’s scale^(24)^ as follows: 0–0.2 = trivial, 0.2–0.6 = small, 0.6–1.2 = moderate, 1.2–2.0 = large, >2.0 = very large. Statistical significance was defined as *p*<0.05.

## Results

### Participant flow and baseline characteristics

**Fig. 1:**
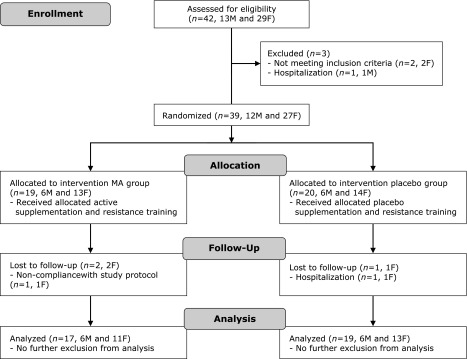
CONSORT Flow diagram. Flowchart of participant recruitment and dropouts before and during the study. F, female participant; M, Male participant.

A Consolidated Standards of Reporting Trials flow diagram of the study is provided in Fig. 1. Thirty-nine participants fulfilled the inclusion criteria and were randomly assigned to the MA (*n* = 19) or the placebo group (*n* = 20). Two female participants in the MA group left the study during the intervention period owing to hospitalization and difficulties in complying with the study protocol, respectively. One female participant in the placebo group dropped out during the intervention period owing to hospitalization. Total 36 (92.3%) participants (MA group, *n* = 17, 89.5%; placebo group, *n* = 19, 95.0%) completed the 12-week intervention study. During the study, no adverse effects were reported following the administration with the MA or the placebo supplements. The baseline characteristics are shown in Table 1. No significant differences were noted between the groups at baseline. According to the MNA and dietary food records before intervention, no participant was malnourished. Moreover, none of the participant consumed commercial supplements including maslinic- or ursolic-acid before and during the study.

### Compliance

The participants with both the groups, compliance with assigned study supplementation was high (MA group, 99.5 ± 1.4%; placebo group, 99.2 ± 1.3%, *p* = 0.56) during the 12-week intervention period. In both the groups, participants performed similar training in terms of the percentage of RT classes attended and the frequency of performing home exercise (MA group, 74.1 ± 25.3%; placebo group, 83.3 ± 14.5%, *p* = 0.20).

### Mobility-related parameters

Table 2 shows the body composition and mobility-related parameters including knee pain scores for the MA and the placebo groups at baseline and at 12 weeks.

The participants of the MA group showed a significant decrease in percentage of fat mass (*p* = 0.011, effect size Cohen’s *d* = 0.71), a tendency to increase in skeletal muscle mass (*p* = 0.061, *d* = 0.47), and a significant increase in the muscle mass of the right arm (*p*<0.001, *d* = 1.34), left arm (*p* = 0.005, *d* = 0.84) and trunk (*p*<0.001, *d* = 1.4), while the placebo group participants did not differ in any body composition values between baseline and 12 weeks. These changes in skeletal muscle mass (group × time interaction, *F*_1, 34_ = 4.98, *p* = 0.032), segmented muscle mass of the right arm (*F*_1, 34_ = 12.72, *p* = 0.001), left arm (*F*_1, 34_ = 7.22, *p* = 0.011), and trunk (*F*_1, 34_ = 11.84, *p* = 0.002) were significantly greater than in the placebo group, respectively.

For physical performance, indicators such as muscle strength for individuals in the MA group showed a significant increase in grip strength (better side) from baseline (*p* = 0.025, *d* = 0.60), while an increase in the placebo group did not reach statistical significance (*p* = 0.20, *d* = 0.31). The timed 5-meter walk was significantly increased in the both the MA [*p* = 0.015 (*p* = 0.025, *d* = 0.72)] and the placebo groups (*p* = 0.003, *d* = 0.79), indicating that participants of both the groups walked at a slower pace compared to baseline. Times of stand-up from a chair and the mobility function score did not differ between baseline and 12 weeks in both the groups.

The level of knee pain was assessed by using the osteoarthritis index, WOMAC,^(22)^ whose higher scores represent better function or less pain. In both the groups, all parameter (total, knee function, right knee pain and left knee pain) scores did not significantly differ between baseline and 12 weeks, however, the repeated measurement ANOVA showed that the knee pain score of the right leg in the MA group was more improved than in the placebo group (group × time interaction, *F*_1, 34_ = 4.58, *p* = 0.040).

## Discussion

This 12-week trial for aged, community-dwelling individuals demonstrated new intriguing findings. Compared to placebo, the MA supplements with moderate RT may improve body composition by increasing upper-body muscle mass, grip strength of the better side, and knee pain of the right leg.

After intervention, no change was noted in the body weight or muscle mass of the lower limbs in the MA group, but the whole body skeletal muscle mass increased, body fat percentage decreased, and by region, muscle increased in the upper-body, such as both arms and trunk. Regarding the MA and muscle synthesis, promotion of body protein synthesis,^(25–27)^ and inhibition of loss of muscle through anti-inflammatory actions^(12,28,29)^ have been reported. These are discussed below in the same order. Regarding body protein synthesis, it has been reported that when rainbow trout were fed with a MA-containing diet, the weight of white muscle increased. It has been theorized that MA acts as a growth factor by binding to specific cytoplasmic receptors involved in protein synthesis in order to activate specific protein synthesis-related genes.^(25–27)^ With respect to inhibition of loss of muscle through anti-inflammatory actions, MA treatment significantly decreased release of inflammation-inducing cytokines [interleukin-6 (IL-6) and tumor necrosis factor-α (TNF-α)] from inflammation-induced human mononuclear cells^(28)^ and mouse peritoneal cells.^(29)^ It has also been reported that muscle mass and muscle strength are low in humans with high blood levels of IL-6 and TNF-α.^(30)^ TNF-α induces loss of muscle with aging,^(31)^ and the IL-6 level is high in frail elderly persons.^(32)^ Accordingly, inhibition of an increase in inflammatory cytokines may be involved in the MA-induced increase in muscle mass. However, no inflammation-related biomarker was measured in this study, and this remains to be investigated in the future.

In terms of the loss of muscle mass with aging, the muscle mass of the lower limbs starts to decrease in in people in their 20 s, the reduction rate is higher than that in the upper limbs, and muscle loss accelerates in the senile state,^(26)^ but lower-limb muscle did not decrease in the MA group. A combination of the MA supplements ingestion and safe and practical moderate RT may prevent and improve physical frailty.

Maintenance of muscle strength retains the mobility function, and is important in the QOL of elderly persons, but muscle quality is also an important factor for determining muscle weakness in addition to muscle mass in the elderly individual.^(33)^ In addition, muscle weakness with aging is due to both loss of muscle mass and decline of the output capability of senile muscle.^(34)^ In this study, in the group receiving MA, the grip strength increased in addition to the significant increase in the muscle mass of the upper limbs, suggesting that output capability of the muscles improved. An increase in muscle strength by training is considered to depend on neurological factors determining an increase in the discharge capacity of the motor unit and morphological changes in contractile muscle tissue, i.e., muscular hypertrophy.^(34)^ In the elderly, muscle strength increases mostly through neurological factors, such as an increase in the impulse discharge, throughout the training period.^(34,35)^ Therefore, when considering the increase in grip strength, the contribution of improvement of neurological factors may have been larger than that of muscular hypertrophy.

The walking speed at the usual pace was measured as an index of lower-limb muscle strength, and it slowed after intervention in both groups. Before intervention, the mean speed was 1.7 m/s in both groups, which is faster than that at the population with same age (1.0–1.2 m/s),^(36)^ and this may have been due to the fact that the measurement after intervention was performed in August when the temperature is the highest in the year in Japan.

Three intervention studies on the effect of MA or olive fruit on knee pain improvement in the elderly persons have been performed.^(17,37,38)^ Japanese middle-to-elderly persons (58 ± 7 years) with mild knee pain ingested a capsule containing MA derived from olive fruit (50 mg/day) or placebo capsule for 12 weeks in a study in which significant reduction of body weight loss and improvement of knee pain symptom were observed in the MA group.^(37)^ In the following study reported by the same research group, Japanese elderly persons with knee pain ingested olive fruit jelly (MA: 30 mg/day) for 16 weeks, and the knee pain score improved in the MA group.^(17)^ In another study, a 20-week intervention was conducted in which Japanese elderly women with osteoarthritis of the knee ingested a capsule containing MA derived from olive fruit (50 mg/day) or placebo capsule and underwent concomitant whole-body vibration training; improvement of knee pain and knee muscle strength were observed in participants with severe osteoarthritis in the MA group.^(38)^ Improvement of knee pain observed in the present study is consistent with the above reports,^(17,37,38)^ and we suggest that MA derived from olive fruit (60 mg/day) is effective in also improving slightly knee pain in community-dwelling elderly persons.

RT is directly effective for improving knee pain in elderly persons,^(39,40)^ but no improvement of knee pain was noted in the placebo group treated with RT alone and this may have been due to insufficiency of intensity, frequency, or duration of training. However, although training alone did not reach an effective level, mechanical load accumulation and inflammatory reaction of joint cartilage with aging are involved in the knee pain of the elderly persons, and this combination with RT may have increased the anti-inflammatory action of MA, which has been demonstrated in cell culture^(10,28)^ and in animals.^(11)^ Therefore, daily exercise at a safe level with concomitant MA ingestion may be useful to prevent and improve knee pain in the elderly. In the MA group, improvement of knee pain was observed only in the right knee [effect size Cohen’s *d* = 0.65 (moderate), left knee: *d* = 0.06 (trivial)], and this may have been owing to the fact that the dominant leg is the right in most Japanese^(41)^ and the mechanical burden is smaller than that on the pivoting foot (left) supporting the body weight, so that pain readily improves on the right side.

The present study had three main limitations. First, the statistical power was limited owing to the small sample size. Second, the intervention period was relatively short. Third, blood sampling was not conducted. The main strengths of the study were its randomized controlled trial (RCT) design and the optimal adherence rates (>99%) observed of the two study groups. Despite the limitations, to our knowledge, this is the first RCT to demonstrate the beneficial effects, in aged, community-dwelling individuals, of combined the MA supplementation and moderate RT on upper body muscle mass, and the parameters of mobility functions, such as grip strength and knee pain score. Further studies are warranted to confirm the present findings using the metabolic disposition of MA in plasma and urine samples^(42,43)^ and to elucidate the effect of MA alone on mobility function in older population.

## Conclusions

The present results suggest that, in community-dwelling elderly individuals, the MA supplements combined with moderate RT may have beneficial effects related to upper body muscle mass and mobility function parameters, such as grip strength and knee pain. Further evaluation of the potential benefits of MA supplements alone on the mobility function of older population is warranted.

## Author Contributions

NN and NS were responsible for the study concept and the design, and supervised during the study. NS and KK designed the analysis and interpretation of data. TM helped the clinical setting. NN, SY, AH and SN carried out the data collection and conducted the data analysis. NN wrote the manuscript and had responsibility for all of the content. All the authors reviewed and approved the final manuscript.

## Figures and Tables

**Table 1:** Baseline characteristics of the study participants^†^

Variables	MA group (*n* = 17)	Placebo group (*n* = 19)
No of women	11	13
Age (years)	72.7 ± 1.4	73.5 ± 2.3
Education (years)	11.6 ± 2.3	11.6 ± 1.9
Height (cm)	154.9 ± 5.2	154.0 ± 7.3
Body mass (kg)	52.6 ± 8.2	55.7 ± 11.0
Body mass index (kg/m^2^)	21.9 ± 3.1	23.4 ± 3.7
Systolic blood pressure (mmHg)	140.0 ± 14.0	135.1 ± 18.0
Diastolic blood pressure (mmHg)	76.0 ± 10.0	77.5 ± 10.0
Resting heart rate (bpm)	67.0 ± 11.4	72.3 ± 12.7
Enegy intake (MJ/day)	7.89 ± 2.16	8.37 ± 1.97
Protein intake (g/day)	70.9 ± 20.7	77.8 ± 18.8
Duration of habitual exercise (min/week)	182 ± 140	217 ± 166
Duration of sleep (min/day)	475 ± 75	474 ± 41

Mean values with their standard deviations. MA, maslinic acid. ^†^Unpaired *t* test showed no significant differences at baseline between the two groups.

**Table 2:** Body mass, body composition, segmental muscle mass and mobility related functions

	MA group (*n* = 17)		*p*^†^	Placebo group (*n* = 19)		*p*^†^	*p*^††^
	Baseline	12 weeks		Baseline	12 weeks		
Body mass (kg)	52.6 ± 8.2	52.4 ± 8.5	0.43	55.7 ± 11.0	55.1 ± 10.9	0.049	0.38
Body mass index (kg/m^2^)	21.9 ± 3.1	21.8 ± 3.2	0.48	23.4 ± 3.7	23.1 ± 3.7	0.056	0.37
Body composition							
Fat mass (%)	25.7 ± 6.8	24.6 ± 7.3	0.011	29.0 ± 7.8	28.6 ± 8.0	0.37	0.26
Fat free mass (%)	71.5 ± 8.0	75.4 ± 7.3	0.11	71.0 ± 7.8	74.4 ± 6.8	0.33	0.066
Skeletal muscle mass (kg)	20.9 ± 3.4	21.1 ± 3.4	0.061	21.1 ± 4.3	21.0 ± 4.4	0.25	0.032
Segmental muscle mass							
Right arm (kg)	1.89 ± 0.44	1.95 ± 0.46	<0.001	1.92 ± 0.52	1.91 ± 0.54	0.74	0.001
Left arm (kg)	1.86 ± 0.42	1.90 ± 0.44	0.005	1.89 ± 0.53	1.86 ± 0.54	0.19	0.011
Trunk (kg)	17.05 ± 2.61	17.30 ± 2.69	<0.001	17.17 ± 3.33	17.09 ± 3.38	0.31	0.002
Right leg (kg)	5.75 ± 0.99	5.73 ± 1.05	0.74	5.78 ± 1.26	5.73 ± 1.25	0.45	0.74
Left leg (kg)	5.74 ± 0.98	5.75 ± 1.06	0.96	5.76 ± 1.24	5.76 ± 1.24	0.96	1.00
Physical performance							
Grip strength (better side, kg)	24.3 ± 7.4	25.7 ± 7.2	0.025	23.2 ± 7.4	24.1 ± 7.3	0.20	0.57
Timed 5-m walk (s)	2.90 ± 0.26	3.04 ± 0.25	0.015	3.00 ± 0.34	3.17 ± 0.37	0.003	0.66
Stand-up from chair (times/30 s)	17.4 ± 2.2	18.2 ± 3.9	0.42	18.2 ± 2.9	17.5 ± 3.9	0.20	0.16
Mobility function score (score, 1–5)	1.2 ± 1.1	0.9 ± 0.8	0.26	0.8 ± 1.0	1.0 ± 1.0	0.27	0.11
Osteoarthritis index (WOMAC)							
Total (score)	129.8 ± 5.3	130.4 ± 5.2	0.38	130.4 ± 6.4	130.7 ± 6.2	0.83	0.78
Knee function (score)	82.6 ± 2.6	82.5 ± 3.6	0.59	81.9 ± 4.5	81.7 ± 4.9	0.73	0.45
Pain in right knee (score)	23.6 ± 2.1	24.3 ± 1.2	0.26	24.2 ± 1.6	24.2 ± 1.4	1.00	0.040
Pain in left knee (score)	23.5 ± 1.8	23.6 ± 2.3	0.83	24.2 ± 1.4	24.7 ± 0.6	0.14	0.56

Mean values with their standard deviations. MA, maslinic acid. WOMAC, Western Ontario and McMaster Universities osteoarthritis index. Higher scores represent better situation or less pain. ^†^*P* value obtained from paired *t* test to compare changes from baseline assessment (baseline vs 12 weeks) within the MA and the placebo groups. ^††^*P* value represents between-group comparison of changes from baseline assessment (baseline vs 12 weeks) in repeated measurement ANOVA assessing group × time interaction.

## Abbreviations

MA: maslinic acid
QOL: quality of life
RCT: randomized controlled trial
RT: resistance training
